# The genome sequence of the Common Wainscot moth, *Mythimna pallens *Linnaeus, 1758

**DOI:** 10.12688/wellcomeopenres.23320.1

**Published:** 2024-11-06

**Authors:** Stephanie Holt, Laura Sivess, Inez Januszczak, Gavin R. Broad, Chris Fletcher, Natural History Museum Genome Acquisition Lab, Darwin Tree of Life Barcoding collective, Wellcome Sanger Institute Tree of Life Management, Samples and Laboratory team, Wellcome Sanger Institute Scientific Operations: Sequencing Operations, Wellcome Sanger Institute Tree of Life Core Informatics team, Tree of Life Core Informatics collective, Darwin Tree of Life Consortium

**Affiliations:** 1 Natural History Museum, London, England, UK

**Keywords:** Mythimna pallens, Common Wainscot moth, genome sequence, chromosomal, Lepidoptera

## Abstract

We present a genome assembly from an individual male *Mythimna pallens* (the Common Wainscot moth; Arthropoda; Insecta; Lepidoptera; Noctuidae). The genome sequence has a total length of 719.10 megabases. Most of the assembly is scaffolded into 31 chromosomal pseudomolecules, including the Z sex chromosome. The mitochondrial genome has also been assembled and is 15.33 kilobases in length. Gene annotation of this assembly on Ensembl identified 18,343 protein-coding genes.

## Species taxonomy

Eukaryota; Opisthokonta; Metazoa; Eumetazoa; Bilateria; Protostomia; Ecdysozoa; Panarthropoda; Arthropoda; Mandibulata; Pancrustacea; Hexapoda; Insecta; Dicondylia; Pterygota; Neoptera; Endopterygota; Amphiesmenoptera; Lepidoptera; Glossata; Neolepidoptera; Heteroneura; Ditrysia; Obtectomera; Noctuoidea; Noctuidae; Hadeninae; *Mythimna*; *Mythimna pallens* Linnaeus, 1758 (NCBI:txid987986).

## Background

The Common Wainscot *Mythimna pallens* has a broad distribution across most of the British Isles, although becoming less common towards the far north of Scotland ( NBN Atlas Partnership, 2024). Towards the northernmost limits of its distribution, it is only single brooded, with a flight period between July and August, however further south it has two generations and can be found on the wing between May and October. Globally it ranges across the Palaearctic region, from Ireland to Russia and into central Asia.

Adult colour can range from pale straw to reddish brown, with pale forewing veins. A postmedian line of black dots may be present. The species has a wingspan of 32–40 mm and a forewing length of 14–17 mm ( Waring *et al.*, 2017). It may be confused with *M. favicolor, M. impura* and *M. straminea*, however *M. pallens* can be differentiated by a narrower forewing base and a sharply acute angle between the termen and the costa. Genitalia in both male and female again show high degrees of similarity between *M. pallens*, *M. impura* and *M. straminea* ( Lewis, 2018). The larvae are yellow to reddish with a dorsal white line with a black edge. Black spiracles are situated within a yellow-white lateral stripe ( Townsend *et al.*, 2010). The larvae feed on a variety of grass species including Deschampsia, Festuca, Leymus, Lolium and Phalaris ( Robinson *et al.*, 2010). The species overwinters as a larva.

This specimen was captured in a light trap at the Gilbert White House & Museum in Selborne, near Alton, Hampshire, during a genome-blitz for the Darwin Tree of Life project by a team from the Natural History Museum. Gilbert White (1720–1793) was a pioneer in observational natural history and commonly held to be the ‘father of ecology’. He is famed for his *Natural History and Antiquities of Selborne* ( White, 1789) which highlighted the depths of his studies in his home village, particularly in his garden from which this specimen was taken. This species has been recorded regularly at the site since moth recording began in 2016 and has been found in all trapping events during June-September, with the highest count in August 2022 (36 individuals).

## Genome sequence report

**Figure 1.**
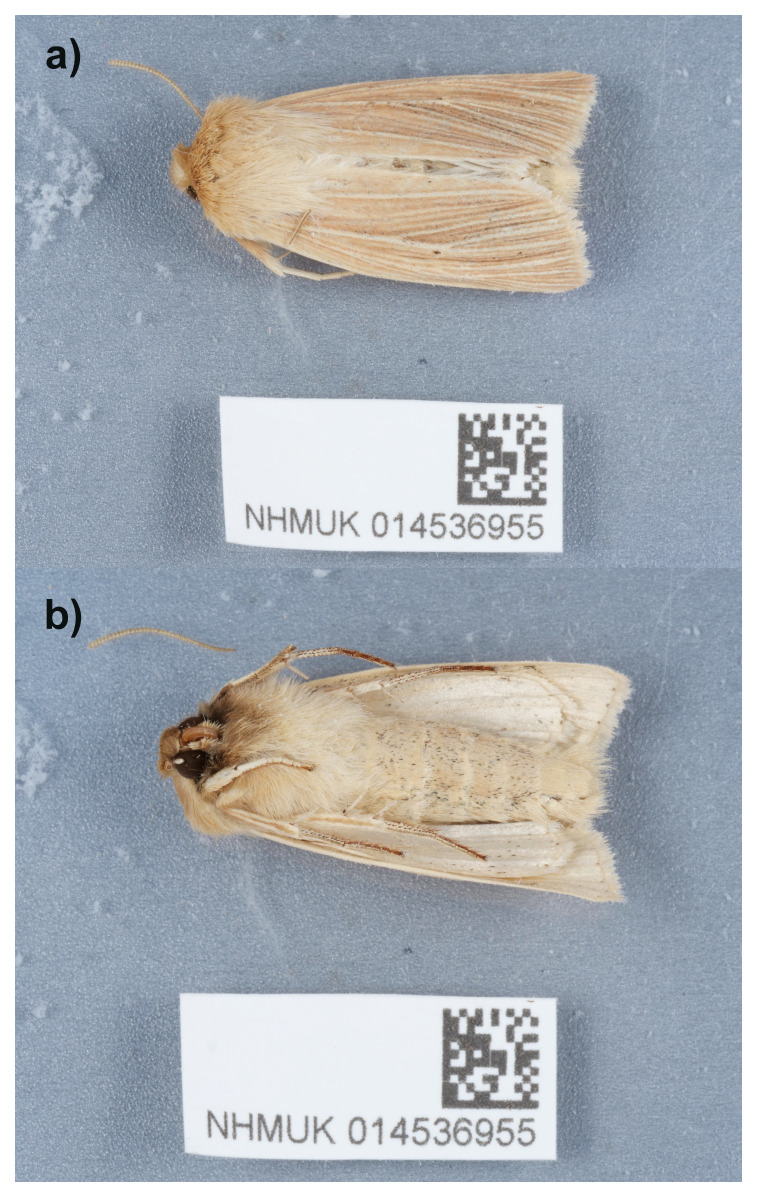
Photograph of the *Mythimna pallens* (ilMytPall1) specimen used for genome sequencing.

The genome of an adult male specimen of *Mythimna pallens* ( Figure 1) was sequenced using Pacific Biosciences single-molecule HiFi long reads, generating a total of 27.49 Gb (gigabases) from 2.09 million reads, providing approximately 36-fold coverage. Primary assembly contigs were scaffolded with chromosome conformation Hi-C data, which produced 119.17 Gb from 789.23 million reads. Specimen and sequencing details are summarised in Table 1.

**Table 1.** Specimen and sequencing data for *Mythimna pallens*.

Project information			
**Study title**	Mythimna pallens		
**Umbrella BioProject**	PRJEB62620		
**Species**	*Mythimna pallens*		
**BioSample**	SAMEA112222349		
**NCBI taxonomy ID**	987986		
Specimen information			
**Technology**	**ToLID**	**BioSample accession**	**Organism part**
**PacBio long read sequencing**	ilMytPall1	SAMEA112222424	thorax
**Hi-C sequencing**	ilMytPall1	SAMEA112222421	head
**RNA sequencing**	ilMytPall1	SAMEA112222419	abdomen
Sequencing information			
**Platform**	**Run accession**	**Read count**	**Base count (Gb)**
**Hi-C Illumina NovaSeq 6000**	ERR11496095	7.89e+08	119.17
**PacBio Sequel IIe**	ERR11483524	2.09e+06	27.49
**RNA Illumina NovaSeq X**	ERR12862078	6.49e+07	9.79

**Figure 2.**
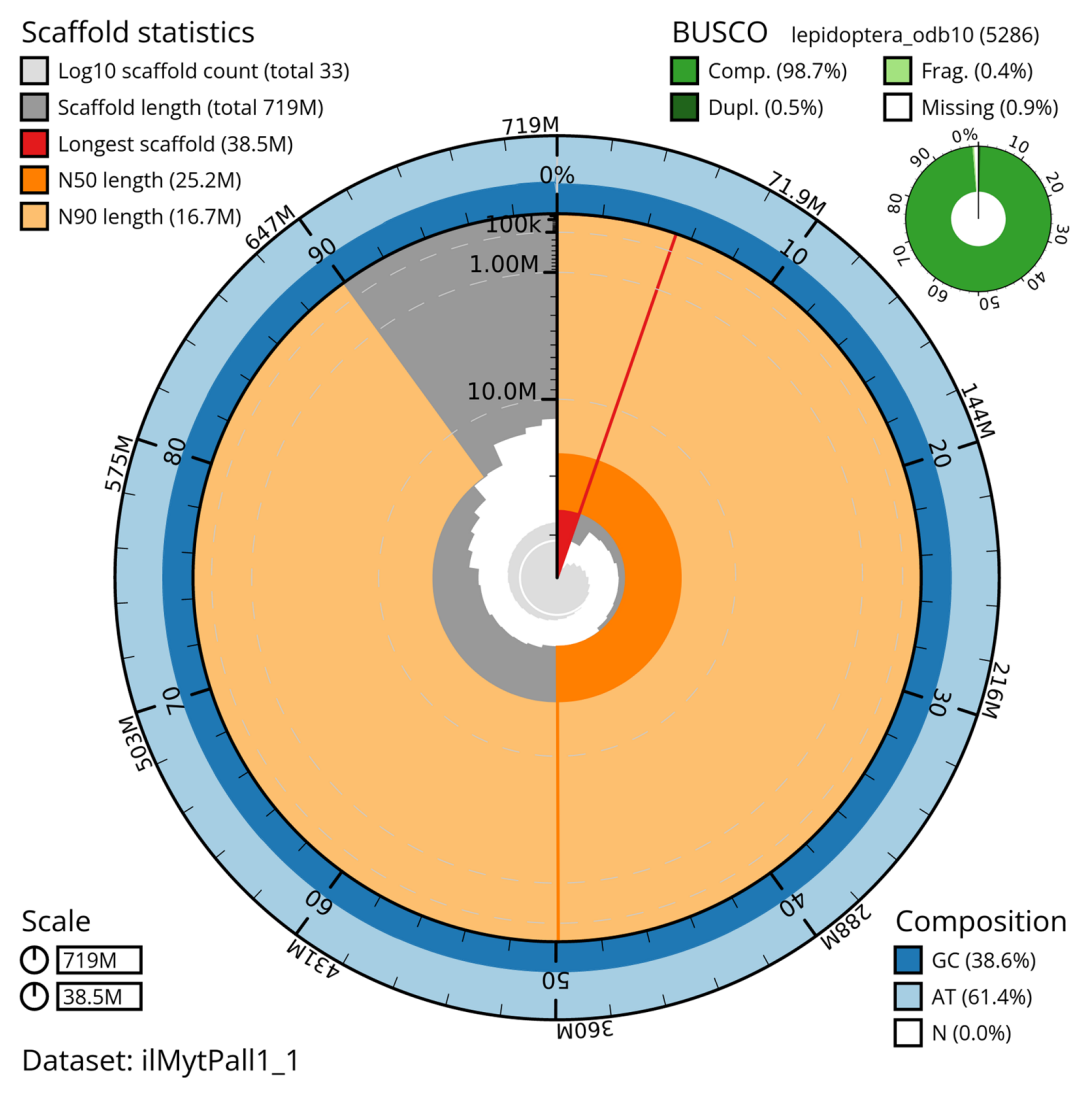
Genome assembly of *Mythimna pallens*, ilMytPall1.1: metrics. The BlobToolKit snail plot shows N50 metrics and BUSCO gene completeness. The main plot is divided into 1,000 size-ordered bins around the circumference with each bin representing 0.1% of the 719,100,281 bp assembly. The distribution of scaffold lengths is shown in dark grey with the plot radius scaled to the longest scaffold present in the assembly (38,484,537 bp, shown in red). Orange and pale-orange arcs show the N50 and N90 scaffold lengths (25,192,387 and 16,654,780 bp), respectively. The pale grey spiral shows the cumulative scaffold count on a log scale with white scale lines showing successive orders of magnitude. The blue and pale-blue area around the outside of the plot shows the distribution of GC, AT and N percentages in the same bins as the inner plot. A summary of complete, fragmented, duplicated and missing BUSCO genes in the lepidoptera_odb10 set is shown in the top right. An interactive version of this figure is available at https://blobtoolkit.genomehubs.org/view/ilMytPall1_1/dataset/ilMytPall1_1/snail.

**Figure 3.**
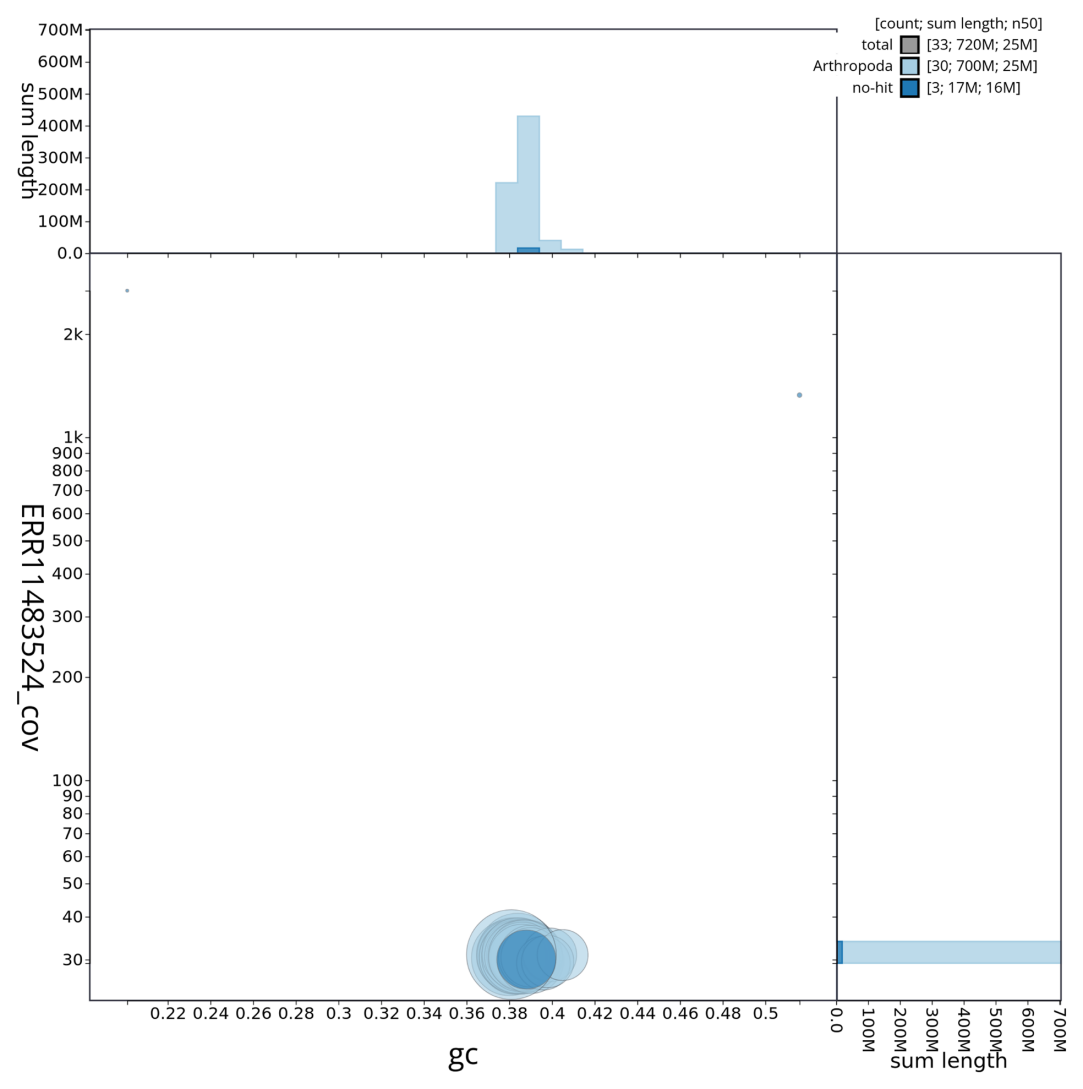
Genome assembly of *Mythimna pallens*, ilMytPall1.1: BlobToolKit GC-coverage plot showing sequence coverage (vertical axis) and GC content (horizontal axis). The circles represent scaffolds, with the size proportional to scaffold length and the colour representing phylum membership. The histograms along the axes display the total length of sequences distributed across different levels of coverage and GC content. An interactive version of this figure is available at https://blobtoolkit.genomehubs.org/view/ilMytPall1_1/dataset/ilMytPall1_1/blob.

**Figure 4.**
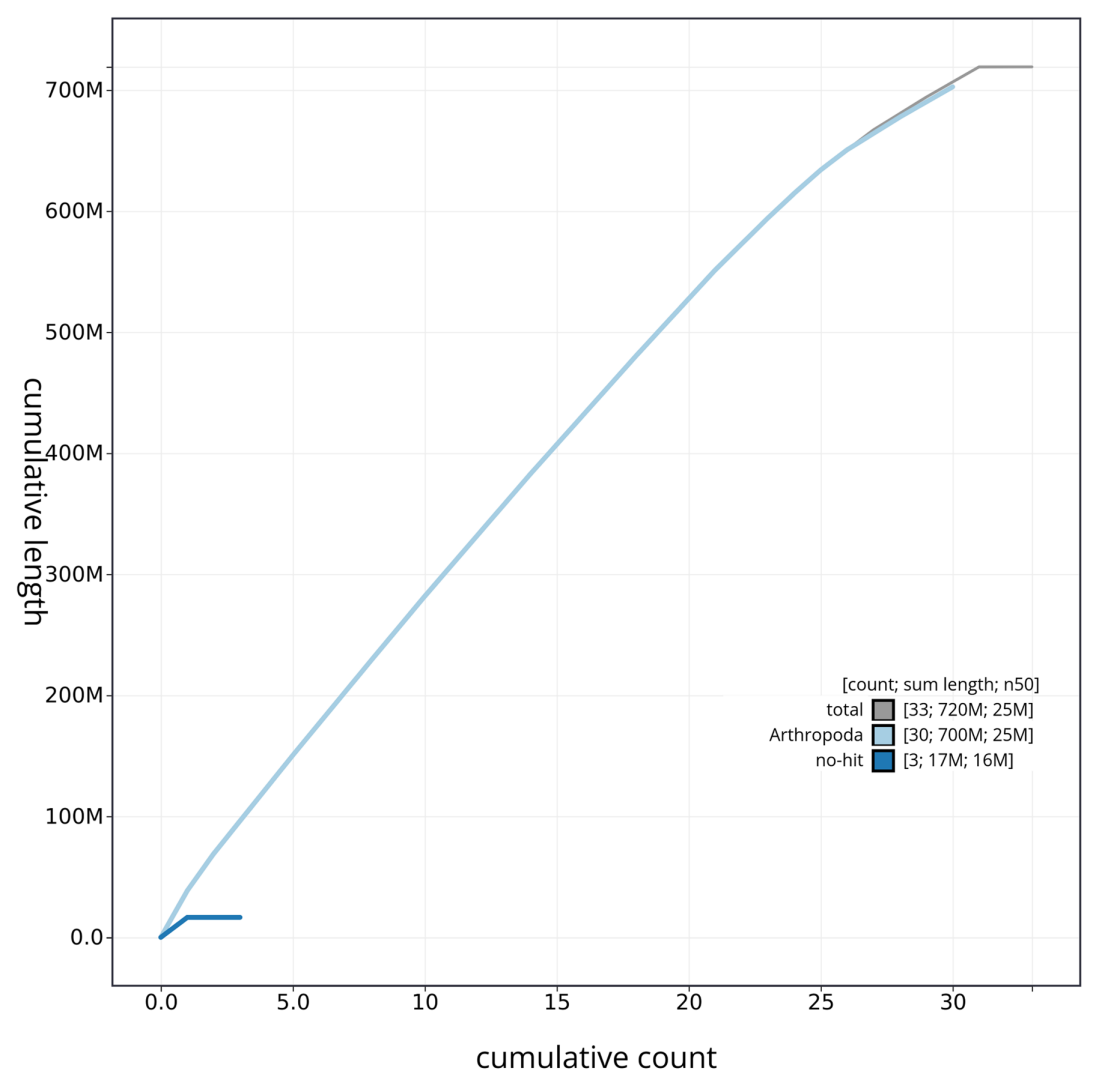
Genome assembly of *Mythimna pallens* ilMytPall1.1: BlobToolKit cumulative sequence plot. The grey line shows cumulative length for all sequences. Coloured lines show cumulative lengths of sequences assigned to each phylum using the buscogenes taxrule. An interactive version of this figure is available at https://blobtoolkit.genomehubs.org/view/ilMytPall1_1/dataset/ilMytPall1_1/cumulative.

**Figure 5.**
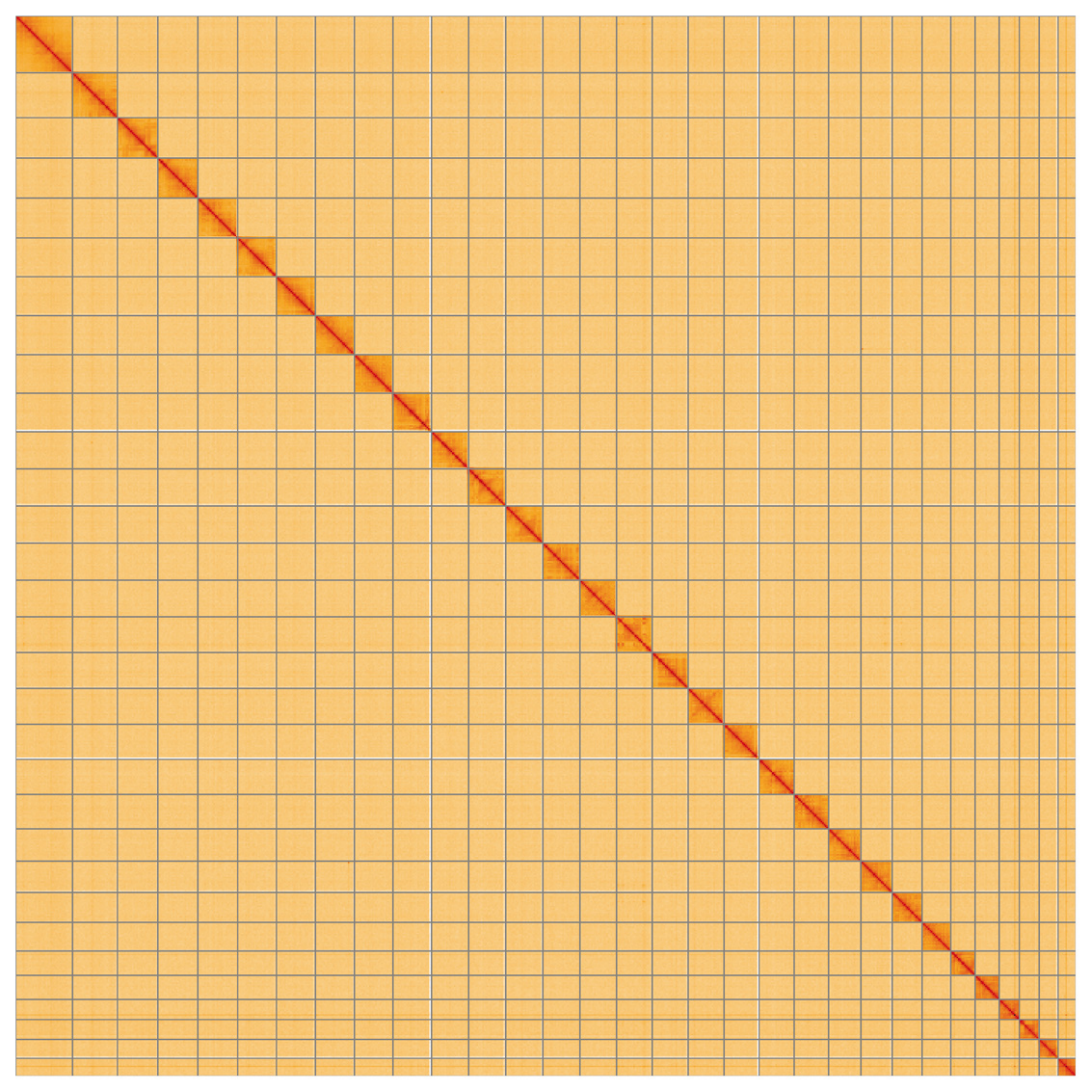
Genome assembly of *Mythimna pallens*, ilMytPall1.1: Hi-C contact map of the ilMytPall1.1 assembly, visualised using HiGlass. Chromosomes are shown in order of size from left to right and top to bottom. An interactive version of this figure may be viewed at https://genome-note-higlass.tol.sanger.ac.uk/l/?d=OQkjDGb1SMa5ATHrdz-Ipg.

Manual assembly curation corrected 13 missing joins or mis-joins and two haplotypic duplications, reducing the assembly length by 0.91%. The final assembly has a total length of 719.10 Mb in 32 sequence scaffolds with a scaffold N50 of 25.2 Mb ( Table 2). The total count of gaps in the scaffolds is 64. The snail plot in Figure 2 provides a summary of the assembly statistics, while the distribution of assembly scaffolds on GC proportion and coverage is shown in Figure 3. The cumulative assembly plot in Figure 4 shows curves for subsets of scaffolds assigned to different phyla. Most (99.99%) of the assembly sequence was assigned to 31 chromosomal-level scaffolds, representing 30 autosomes and the Z sex chromosome. Chromosome-scale scaffolds confirmed by the Hi-C data are named in order of size ( Figure 5; Table 3). Chromosome Z was assigned by synteny to *Mythimna l-album* (GCA_949319445.1) ( Sterling *et al.*, 2024). While not fully phased, the assembly deposited is of one haplotype. Contigs corresponding to the second haplotype have also been deposited. The mitochondrial genome was also assembled and can be found as a contig within the multifasta file of the genome submission.

**Table 2.** Genome assembly data for *Mythimna pallens*, ilMytPall1.1.

Genome assembly		
Assembly name	ilMytPall1.1	
Assembly accession	GCA_961205895.1	
*Accession of alternate haplotype*	*GCA_961205865.1*	
Span (Mb)	719.10	
Number of contigs	97	
Number of scaffolds	32	
Longest scaffold (Mb)	38.48	
Assembly metrics *		*Benchmark*
Contig N50 length (Mb)	14.4	*≥ 1 Mb*
Scaffold N50 length (Mb)	25.2	*= chromosome N50*
Consensus quality (QV)	69.5	*≥ 40*
*k*-mer completeness	100.0%	*≥ 95%*
BUSCO **	C:98.7%[S:98.2%,D:0.5%], F:0.4%,M:0.9%,n:5,286	*S > 90%* *D < 5%*
Percentage of assembly mapped to chromosomes	99.99%	*≥ 90%*
Sex chromosomes	Z	*localised homologous pairs*
Organelles	Mitochondrial genome: 15.33 kb	*complete single alleles*
Genome annotation of assembly GCA_961205895.1 at Ensembl		
Number of protein-coding genes	18,343	
Number of gene transcripts	18,527	

* Assembly metric benchmarks are adapted from Rhie *et al.* (2021) and the Earth BioGenome Project Report on Assembly Standards September 2024. ** BUSCO scores based on the lepidoptera_odb10 BUSCO set using version 5.3.2. C = complete [S = single copy, D = duplicated], F = fragmented, M = missing, n = number of orthologues in comparison. A full set of BUSCO scores is available at https://blobtoolkit.genomehubs.org/view/ilMytPall1_1/dataset/ilMytPall1_1/busco.

**Table 3.** Chromosomal pseudomolecules in the genome assembly of *Mythimna pallens*, ilMytPall1.

INSDC accession	Name	Length (Mb)	GC%
OY540825.1	1	30.54	38.5
OY540826.1	2	27.39	38.5
OY540827.1	3	27.13	38.0
OY540828.1	4	26.9	38.5
OY540829.1	5	26.48	38.5
OY540830.1	6	26.39	38.5
OY540831.1	7	26.35	38.5
OY540832.1	8	26.21	38.0
OY540833.1	9	25.93	38.5
OY540834.1	10	25.31	38.0
OY540835.1	11	25.21	38.5
OY540836.1	12	25.21	38.5
OY540837.1	13	25.19	38.5
OY540838.1	14	24.69	38.5
OY540839.1	15	24.4	39.0
OY540840.1	16	24.27	38.5
OY540841.1	17	24.15	39.0
OY540842.1	18	23.75	38.5
OY540843.1	19	23.75	38.5
OY540844.1	20	23.48	38.5
OY540845.1	21	21.9	38.5
OY540846.1	22	21.12	38.5
OY540847.1	23	20.42	39.0
OY540848.1	24	19.26	39.0
OY540849.1	25	16.65	39.0
OY540850.1	26	16.46	39.0
OY540851.1	27	13.53	40.0
OY540852.1	28	13.54	39.5
OY540853.1	29	12.82	40.0
OY540854.1	30	12.11	40.5
OY540824.1	Z	38.48	38.0
OY540855.1	MT	0.02	20.5

The estimated Quality Value (QV) of the final assembly is 69.5 with *k*-mer completeness of 100.0%, and the assembly has a BUSCO v5.3.2 completeness of 98.7% (single = 98.2%, duplicated = 0.5%), using the lepidoptera_odb10 reference set ( *n* = 5,286).

Metadata for specimens, BOLD barcode results, spectra estimates, sequencing runs, contaminants and pre-curation assembly statistics are given at https://links.tol.sanger.ac.uk/species/987986.

## Genome annotation report

The *Mythimna pallens* genome assembly (GCA_961205895.1) was annotated at the European Bioinformatics Institute (EBI) on Ensembl Rapid Release. The resulting annotation includes 18,527 transcribed mRNAs from 18,343 protein-coding genes ( Table 2; https://rapid.ensembl.org/Mythimna_pallens_GCA_961205895.1/Info/Index). The average transcript length is 8,149.37, with 5.39 exons per transcript.

## Methods

### Sample acquisition and DNA barcoding

An adult *Mythimna pallens* (specimen ID NHMUK014536955, ToLID ilMytPall1) was collected from Gilbert White’s House, Selborne, UK (latitude 51.09, longitude –0.94) on 2021-06-10, using a light trap. The specimen was collected by Inez Januszczak, Gavin Broad, Laura Sivess, Stephanie Holt and Chris Fletcher (Natural History Museum) and identified by Stephanie Holt, and then preserved by dry freezing at –80 °C.

The initial identification was verified by an additional DNA barcoding process according to the framework developed by Twyford *et al*. (2024). A small sample was dissected from the specimen and stored in ethanol, while the remaining parts of the specimen were shipped on dry ice to the Wellcome Sanger Institute (WSI). The tissue was lysed, the COI marker region was amplified by PCR, and amplicons were sequenced and compared to the BOLD database, confirming the species identification ( Crowley *et al.*, 2023). Following whole genome sequence generation, the relevant DNA barcode region is also used alongside the initial barcoding data for sample tracking at the WSI ( Twyford *et al.*, 2024). The standard operating procedures for Darwin Tree of Life barcoding have been deposited on protocols.io ( Beasley *et al.*, 2023).

### Nucleic acid extraction

The workflow for high molecular weight (HMW) DNA extraction at the WSI Tree of Life Core Laboratory includes a sequence of core procedures: sample preparation and homogenisation, DNA extraction, fragmentation and purification. Detailed protocols are available on protocols.io ( Denton *et al.*, 2023b). The ilMytPall1 sample was weighed and dissected on dry ice ( Jay *et al.*, 2023), and tissue from the thorax was homogenised using a PowerMasher II tissue disruptor ( Denton *et al.*, 2023a).

HMW DNA was extracted at the WSI Scientific Operations core using the Automated MagAttract v2 protocol ( Oatley *et al.*, 2023). The DNA was sheared into an average fragment size of 12–20 kb in a Megaruptor 3 system ( Bates *et al.*, 2023). Sheared DNA was purified by solid-phase reversible immobilisation, using AMPure PB beads to eliminate shorter fragments and concentrate the DNA ( Strickland *et al.*, 2023). The concentration of the sheared and purified DNA was assessed using a Nanodrop spectrophotometer and Qubit Fluorometer using the Qubit dsDNA High Sensitivity Assay kit. Fragment size distribution was evaluated by running the sample on the FemtoPulse system.

RNA was extracted from abdomen tissue of ilMytPall1 in the Tree of Life Laboratory at the WSI using the RNA Extraction: Automated MagMax™ *mir*Vana protocol ( do Amaral *et al.*, 2023). The RNA concentration was assessed using a Nanodrop spectrophotometer and a Qubit Fluorometer using the Qubit RNA Broad-Range Assay kit. Analysis of the integrity of the RNA was done using the Agilent RNA 6000 Pico Kit and Eukaryotic Total RNA assay.

### Hi-C preparation

Head tissue of the ilMytPall1sample was processed at the WSI Scientific Operations core, using the Arima-HiC v2 kit. Tissue (stored at –80 °C) was fixed, and the DNA crosslinked using a TC buffer with 22% formaldehyde. After crosslinking, the tissue was homogenised using the Diagnocine Power Masher-II and BioMasher-II tubes and pestles. Following the kit manufacturer's instructions, crosslinked DNA was digested using a restriction enzyme master mix. The 5’-overhangs were then filled in and labelled with biotinylated nucleotides and proximally ligated. An overnight incubation was carried out for enzymes to digest remaining proteins and for crosslinks to reverse. A clean up was performed with SPRIselect beads prior to library preparation.

### Library preparation and sequencing

Library preparation and sequencing were performed at the WSI Scientific Operations core. Pacific Biosciences HiFi circular consensus DNA sequencing libraries were prepared using the PacBio Express Template Preparation Kit v2.0 (Pacific Biosciences, California, USA) as per the manufacturer's instructions. The kit includes the reagents required for removal of single-strand overhangs, DNA damage repair, end repair/A-tailing, adapter ligation, and nuclease treatment. Library preparation also included a library purification step using AMPure PB beads (Pacific Biosciences, California, USA) and size selection step to remove templates shorter than 3 kb using AMPure PB modified SPRI. DNA concentration was quantified using the Qubit Fluorometer v2.0 and Qubit HS Assay Kit and the final library fragment size analysis was carried out using the Agilent Femto Pulse Automated Pulsed Field CE Instrument and gDNA 165kb gDNA and 55kb BAC analysis kit. Samples were sequenced using the Sequel IIe system (Pacific Biosciences, California, USA). The concentration of the library loaded onto the Sequel IIe was in the range 40–135 pM. The SMRT link software, a PacBio web-based end-to-end workflow manager, was used to set-up and monitor the run, as well as perform primary and secondary analysis of the data upon completion.

For Hi-C library preparation, DNA was fragmented to a size of 400 to 600 bp using a Covaris E220 sonicator. The DNA was then enriched, barcoded, and amplified using the NEBNext Ultra II DNA Library Prep Kit following manufacturers’ instructions. The Hi-C sequencing was performed using paired-end sequencing with a read length of 150 bp on an Illumina NovaSeq 6000 instrument.

Poly(A) RNA-Seq libraries were constructed using the NEB Ultra II RNA Library Prep kit, following the manufacturer’s instructions. RNA sequencing was performed on the Illumina NovaSeq X instrument.

### Genome assembly, curation and evaluation

***Assembly***

The HiFi reads were first assembled using Hifiasm ( Cheng *et al.*, 2021) with the --primary option. Haplotypic duplications were identified and removed using purge_dups ( Guan *et al.*, 2020). The Hi-C reads were mapped to the primary contigs using bwa-mem2 ( Vasimuddin *et al.*, 2019). The contigs were further scaffolded using the provided Hi-C data ( Rao *et al.*, 2014) in YaHS ( Zhou *et al.*, 2023) using the --break option. The scaffolded assemblies were evaluated using Gfastats ( Formenti *et al.*, 2022), BUSCO ( Manni *et al.*, 2021) and MERQURY.FK ( Rhie *et al.*, 2020).

The mitochondrial genome was assembled using MitoHiFi ( Uliano-Silva *et al.*, 2023), which runs MitoFinder ( Allio *et al.*, 2020) and uses these annotations to select the final mitochondrial contig and to ensure the general quality of the sequence.

***Assembly curation***

The assembly was decontaminated using the Assembly Screen for Cobionts and Contaminants (ASCC) pipeline (article in preparation). Manual curation was primarily conducted using PretextView ( Harry, 2022), with additional insights provided by JBrowse2 ( Diesh *et al.*, 2023) and HiGlass ( Kerpedjiev *et al.*, 2018). Scaffolds were visually inspected and corrected as described by Howe *et al*. (2021). Any identified contamination, missed joins, and mis-joins were corrected, and duplicate sequences were tagged and removed. The entire process is documented at https://gitlab.com/wtsi-grit/rapid-curation (article in preparation).

***Evaluation of the final assembly***

A Hi-C map for the final assembly was produced using bwa-mem2 ( Vasimuddin *et al.*, 2019) in the Cooler file format ( Abdennur & Mirny, 2020). To assess the assembly metrics, the *k*-mer completeness and QV consensus quality values were calculated in Merqury ( Rhie *et al.*, 2020). This work was done using the “sanger-tol/readmapping” ( Surana *et al.*, 2023a) and “sanger-tol/genomenote” ( Surana *et al.*, 2023b) pipelines. The genome evaluation pipelines were developed using nf-core tooling ( Ewels *et al.*, 2020) and MultiQC ( Ewels *et al.*, 2016), relying on the Conda package manager, the Bioconda initiative ( Grüning *et al.*, 2018), the Biocontainers infrastructure ( da Veiga Leprevost *et al.*, 2017), as well as the Docker ( Merkel, 2014) and Singularity ( Kurtzer *et al.*, 2017) containerisation solutions.

The genome was also analysed within the BlobToolKit environment ( Challis *et al.*, 2020) and BUSCO scores ( Manni *et al.*, 2021) were calculated.

Table 4 contains a list of relevant software tool versions and sources.

**Table 4.** Software tools: versions and sources.

Software tool	Version	Source
BlobToolKit	4.2.1	https://github.com/blobtoolkit/blobtoolkit
BUSCO	5.3.2	https://gitlab.com/ezlab/busco
bwa-mem2	2.2.1	https://github.com/bwa-mem2/bwa-mem2
Gfastats	1.3.6	https://github.com/vgl-hub/gfastats
Hifiasm	0.16.1-r375	https://github.com/chhylp123/hifiasm
HiGlass	44086069ee7d4d3f6f3f0012569789ec138f42b84 aa44357826c0b6753eb28de	https://github.com/higlass/higlass
Merqury.FK	d00d98157618f4e8d1a9190026b19b471055b22e	https://github.com/thegenemyers/MERQURY.FK
MitoHiFi	2	https://github.com/marcelauliano/MitoHiFi
PretextView	0.2	https://github.com/wtsi-hpag/PretextView
purge_dups	1.2.3	https://github.com/dfguan/purge_dups
sanger-tol/genomenote	v1.0	https://github.com/sanger-tol/genomenote
sanger-tol/readmapping	1.1.0	https://github.com/sanger-tol/readmapping/tree/1.1.0
YaHS	yahs-1.1.91eebc2	https://github.com/c-zhou/yahs

### Genome annotation

The BRAKER2 pipeline ( Brůna *et al.*, 2021) was used in the default protein mode to generate annotation for the *Mythimna pallens* assembly (GCA_961205895.1) in Ensembl Rapid Release at the EBI.

### Wellcome Sanger Institute – Legal and Governance

The materials that have contributed to this genome note have been supplied by a Darwin Tree of Life Partner. The submission of materials by a Darwin Tree of Life Partner is subject to the **‘Darwin Tree of Life Project Sampling Code of Practice’**, which can be found in full on the Darwin Tree of Life website here. By agreeing with and signing up to the Sampling Code of Practice, the Darwin Tree of Life Partner agrees they will meet the legal and ethical requirements and standards set out within this document in respect of all samples acquired for, and supplied to, the Darwin Tree of Life Project.

Further, the Wellcome Sanger Institute employs a process whereby due diligence is carried out proportionate to the nature of the materials themselves, and the circumstances under which they have been/are to be collected and provided for use. The purpose of this is to address and mitigate any potential legal and/or ethical implications of receipt and use of the materials as part of the research project, and to ensure that in doing so we align with best practice wherever possible. The overarching areas of consideration are:

•   Ethical review of provenance and sourcing of the material

•   Legality of collection, transfer and use (national and international)

Each transfer of samples is further undertaken according to a Research Collaboration Agreement or Material Transfer Agreement entered into by the Darwin Tree of Life Partner, Genome Research Limited (operating as the Wellcome Sanger Institute), and in some circumstances other Darwin Tree of Life collaborators.

## Data Availability

European Nucleotide Archive: *Mythimna pallens*. Accession number PRJEB62620; https://identifiers.org/ena.embl/PRJEB62620. The genome sequence is released openly for reuse. The *Mythimna pallens* genome sequencing initiative is part of the Darwin Tree of Life (DToL) project. All raw sequence data and the assembly have been deposited in INSDC databases. Raw data and assembly accession identifiers are reported in Table 1 and Table 2.

## Author information

Members of the Natural History Museum Genome Acquisition Lab are listed here: https://doi.org/10.5281/zenodo.12159242.

Members of the Darwin Tree of Life Barcoding collective are listed here: https://doi.org/10.5281/zenodo.12158331.

Members of the Wellcome Sanger Institute Tree of Life Management, Samples and Laboratory team are listed here: https://doi.org/10.5281/zenodo.12162482.

Members of Wellcome Sanger Institute Scientific Operations: Sequencing Operations are listed here: https://doi.org/10.5281/zenodo.12165051.

Members of the Wellcome Sanger Institute Tree of Life Core Informatics team are listed here: https://doi.org/10.5281/zenodo.12160324.

Members of the Tree of Life Core Informatics collective are listed here: https://doi.org/10.5281/zenodo.12205391.

Members of the Darwin Tree of Life Consortium are listed here: https://doi.org/10.5281/zenodo.4783558.
