# Supplementary material for: The genome sequence of the Common Wainscot moth, Mythimna pallens Linnaeus, 1758
Source: Wellcome Open Res. 2024 Nov 6;9:654. [Version 1] doi: 10.12688/wellcomeopenres.23320.1 (PMC11624437; doi:10.12688/wellcomeopenres.23320.1)
Supplement: Peer review history

# Reviewer report: Reviewer response for version 1

**DOI:** 10.21956/wellcomeopenres.25703.r111747

**Published:** 2024-12-06

**Authors:** Wai Lok So

**Affiliations:** 1 The Chinese University of Hong Kong, Hong Kong, Hong Kong

The authors successfully sequenced a male *Mythimna pallens *genome, with high completeness and continuity. The sequenced species is commonly found in Britain and the generated resources undoubtedly provided future insight in the study of Lepidoptera. The methods used for animal identification, genome sequencing and analysis was performed neatly in replicable standards. The validation and annotation processes were also performed professionally with clear protocols. The data was also deposited at European Nucleotide Archive (ENA), which is easily accessible to other researchers who are interested.

Are sufficient details of methods and materials provided to allow replication by others?

Yes

Is the rationale for creating the dataset(s) clearly described?

Yes

Are the datasets clearly presented in a useable and accessible format?

Yes

Are the protocols appropriate and is the work technically sound?

Yes

Reviewer Expertise:

NA

I confirm that I have read this submission and believe that I have an appropriate level of expertise to confirm that it is of an acceptable scientific standard.

# Reviewer report: Reviewer response for version 1

**DOI:** 10.21956/wellcomeopenres.25703.r110411

**Published:** 2024-11-26

**Authors:** Jurate De Prins

**Affiliations:** 1 Royal Belgian Institute of Natural Sciences, Brussels, Belgium

Thank you for trusting me to review this article.

 In general, I support, applaud and am happy to see the library of full mitogenomes is growing. Details of molecular methods are not in my comfort zone, but I fully trust the professionalism of co-authors and their supervisors.

 My comments are related to taxonomy and very sincerely wish that taxonomy and molecular data especially presented long read sequences match, and comprehend each other. We all wish that the community of entomologists with every publication goes a step further to enhance our knowledge and build a solid fundament for future research and researchers.

 In this article, it is very clearly stated that “Genitalia in both male and female again show high degrees of similarity between *M. pallens*, *M. impura* and *M. straminea* ( Lewis, 2018).” My logical question is why genitalia images are not presented for the confirmation of species ID? In Lepidoptera, it is a general requirement that species are identified based on multi-character sets. In most cases the ID of species is presented by 1) external morphology; 2) internal morphology (genitalia images); 3) bionomic data; 4) DNA data. All four sets should match, and only then can we be sure what ID is studied by long-read sequences. I strongly recommend including the internal morphology and especially genitalia characters in the protocol. So there will be no confusion among *M. pallens*, *M. impura *and *M. straminea. *The authors have access to the greatest collection in the world, so why not study a historic specimen and have an independent character set for comparison? These are the suggestions to think about.

 The correct species ID for a full mitogenomic data set is crucial. In our super-connected world we have nowadays, accidental species occurrences outside their usual biogeographical area are not exceptions anymore. Tens of such records on moths have been reported in Flanders in recent years (see *Phegea* issues), and I am more than sure that such a pattern is in the UK as well. In the Afrotropical region, we have 118 closely related species of *Mythimna, see https://www.afromoths.net/species?family=&subfamily=&tribe=&scientificname__contains=Mythimna&countries=&plants__family=¶sitoids__family=&plants__scientificname__contains=¶sitoids__scientificname__contains=*

 It is a great probability of accidental import to the UK. As mentioned above, my plea is of more datasets than a habitus photo for species ID.

 If I may, I would encourage the authors to answer the question about the sex-determination mechanism of *M. pallens*. It is very nice to know that the authors determined the presence of the Z chromosome, however, we, readers, are left unaware whether females of this species are ZZ or Z0, since this is important for chromosome conjugation, crossing-over and gene transfer.  

 Thank you for the interesting research and look forward to the next steps.

Are sufficient details of methods and materials provided to allow replication by others?

Yes

Is the rationale for creating the dataset(s) clearly described?

Yes

Are the datasets clearly presented in a useable and accessible format?

Yes

Are the protocols appropriate and is the work technically sound?

Partly

Reviewer Expertise:

Taxonomy, karyology, holistic datasets on moths

I confirm that I have read this submission and believe that I have an appropriate level of expertise to confirm that it is of an acceptable scientific standard.
